# Top health research funders’ guidance on selecting journals for funded research

**DOI:** 10.12688/f1000research.27745.2

**Published:** 2021-04-16

**Authors:** Larissa Shamseer, Kelly D. Cobey, Matthew J. Page, Jamie C. Brehaut, Jeremy M. Grimshaw, Sharon E. Straus, Lesley A. Stewart, David Moher

**Affiliations:** 1 School of Epidemiology and Public Health, University of Ottawa, Ottawa, Ontario, K1G 5Z3, Canada; 2 Li Ka Shing Knowledge Institute, Unity Health Toronto, Toronto, Ontario, M5B 1T8, Canada; 3 Centre for Journalology, Ottawa Hospital Research Institute, Ottawa, ON, K1H 8L6, Canada; 4 Centre for Implementation Research, Ottawa Hospital Research Institute, Ottawa, ON, K1H 8L6, Canada; 5 School of Public Health and Preventive Medicine, Monash University, Melbourne, VIC, 3004, Australia; 6 Department of Medicine, University of Ottawa, Ottawa, Ontario, K1H 8M5, Canada; 7 Centre for Reviews and Dissemination, University of York, UK, York, YO10 5DD, UK

**Keywords:** journals, journal selection, health research funders, publishing

## Abstract

**Background: **Funded health research is being published in journals that many regard as “predatory”, deceptive, and non-credible. We do not currently know whether funders provide guidance on how to select a journal in which to publish funded health research.

**Methods: **We identified the largest 46 philanthropic, public, development assistance, public-private partnership, and multilateral funders of health research by expenditure, globally as well as four public funders from lower-middle income countries, from the list at https://healthresearchfunders.org. One of us identified guidance on disseminating funded research from each funders’ website (August/September 2017), then extracted information about selecting journals, which was verified by another assessor. Discrepancies were resolved by discussion. Results were summarized descriptively. This research used publicly available information; we did not seek verification with funding bodies.

**Results: **The majority (44/50) of sampled funders indicated funding health research. 38 (of 44, 86%) had publicly available information about disseminating funded research, typically called “policies” (29, 76%). Of these 38, 36 (95%) mentioned journal publication for dissemination of which 13 (36.11%) offer variable guidance on selecting a journal, all of which relate to the funder’s open access mandate. Six funders (17%) outlined publisher requirements or features by which to select a journal. One funder linked to a document providing features of journals to look for (e.g. listed in the Directory of Open Access Journals) and to be wary of (e.g., no journal scope statement, uses direct and unsolicited marketing).

**Conclusions: **Few funders provided guidance on how to select a journal in which to publish funded research. Funders have a duty to ensure that the research they fund is discoverable by others. This research is a benchmark for funder guidance on journal selection prior to the January 2021 implementation of Plan S (a global, funder-led initiative to ensure immediate, open access to funded, published research).

## Introduction

Biomedical research studies supported by well-known funding organizations such as the National Institutes of Health (NIH), are published in so-called “predatory” journals ^1^. Predatory journals are regarded as non-credible and are criticized for failing to provide typical or expected publishing services and their lack of transparent operations ^2, 3^. Such services include peer review, long term preservation of content, and indexing in scientific, bibliographic databases. Among their many shortcomings, the potential failure of predatory journals to ensure permanent discoverability of research threatens the integrity of the scientific record. Such research cannot contribute to science, thus wasting time, money, and resources ^1, 4^. Even if discovered, the potential impact and uptake of funded research in predatory journals may be limited due to being published in a perceived untrustworthy source. While benefits from investments in research are difficult to quantify ^5^. One way funders measure returns on investments is by tracking research outputs, including scholarly journal publications ^6^. Predatory journals may limit returns on funders’ investments by undermining the intended promise of scholarly publishing – to enable the results of research to be known for others to build upon ^7^.

Health research funders ought to be concerned that the funds they provide may be wasted or contribute to research waste as a result of funded research being published in predatory journals. They may be supporting research that is not identifiable or able to be found if published in predatory journals, potentially wasting millions of dollars of research funding. When research is easily identifiable it can reduce unintentional redundancies in research efforts and investments. Additional wastage occurs when funder investments are used to pay for article processing charges (APCs). In biomedicine, research grants and national funding agencies are the largest source of funds supporting publication of at least 50% of open access articles ^8^.

### Funders & open access

Most major health research funders mandate that funded research outputs be open access ^9^. Open access mandates typically require researchers to ensure that research (and sometimes data) is published in an open access journal or is deposited in a publicly accessible digital repository (regardless of whether the publication was published in an open access journal), or both. Some journals may impose an embargo period only after which an article is made publicly available or can be archived in a repository (i.e., delayed-access journals). Many funders’ policies allow for such delays in open access to accommodate publishers’ preferences.

Open access policies are one way for funders to direct funded researchers towards publishing in credible journals abiding by established open access tenets ^10^:

1. Research is/should be freely available and accessible to anyone.
2. The copyright accompanying published work should be open, allowing for free use and re-use (i.e., allowing research to be freely built on/adapted with attribution).

To facilitate researcher adherence with funder open access policies, many biomedical journals offering open access have agreements with the PubMed Central (PMC) repository to automatically deposit their published content on authors’ behalf ^11^. Additionally, researchers funded by the NIH and 13 partner funding organizations in the USA can upload funder-supported publications to PMC from journals without PMC agreements ^12^. Likewise, 29 funders from across Europe can submit funder-supported research to Europe PMC (which is mirrored in PMC) ^13^. For some of these organizations, such as the NIH and Wellcome Trust, archiving in PMC or Europe PMC, respectively, is mandatory.

In a possible attempt to attract submissions, predatory journals appear to market themselves as ‘open access’ ^14, 15^. While research in them may indeed be free to access, discovery of their content in scientifically-curated databases is sparse and inconsistent ^16– 18^. Predatory journal articles may haphazardly appear in search engines such as Google Scholar (which indexes anything that appears formatted as a scholarly article) or in PubMed (since it includes author-uploaded articles from PMC) ^19^. Additionally, we do not know whether the contents of unindexed/unarchived journals will be perpetually available if a journal ceases to operate. Such preservation is typically achieved through journal/publisher agreements with digital preservation providers (e.g. Lots of Copies Keep Stuff Safe, LOCKSS). For journals indexed in Medline, for example, this is a prerequisite of indexing ^20^; PMC functions as a preservation service (i.e., has a remit to preserve content funded by public money) ^21^. It is unknown whether predatory journals, not formally indexed in Medline, PMC, or other databases with similar requirements, have digital preservation arrangements.

Most researchers have a limited understanding of what open access means beyond making research free to read ^22– 25^. Free use and unrestricted re-use of research is a fundamental component of open access, and licensing that permits this is a regular component of open access journals ^26^. Journals running nefarious and deceptive publishing operations have likely benefited from or exploited authors’ lack of awareness ^27^. Indeed, few predatory journals mention licensing for articles or provide information on the use and re-use of published research ^26^. Without explicit licensing for published articles, the legalities around distributing or building on research in predatory journals, for example, is uncertain. Whether researchers are deceived by predatory journals or are knowingly seeking easy and rapid publications in them (these journals tend to deliver quicker turnaround time than credible journals due to subpar or non-existent peer review ^28, 29^), they are likely breaching the open access policies set by their funders.

In January 2017, the Bill & Melinda Gates Foundation implemented a policy mandating open access to research publications and data, without delay for all funded research ^30^. In February 2017, they initiated a one-year partnership pilot with the American Association for the Advancement of Science (AAAS) to enable Gates-funded research to be published as open access in five AAAS journals, including *Science* ^31^. The Gates-AAAS partnership seemed to inspire several other influential journals (i.e., *New England Journal of Medicine*, *Proceedings of the National Academy of Sciences*) to introduce policies ensuring permanent open access for Gates-funded research ^32^.

In January 2021, a number of international funders (including UK Research and Innovation, the Gates Foundation, Wellcome Trust, and the World Health Organization), led by Science Europe (a group representing funders across Europe), delivered a radical change to the way that funded research is published, via Plan S ( coalition-s.org). Plan S, in part, requires research funders to mandate open access to funded research through publication in an open access journal or platform; requiring publications to be immediately available through an open access repository upon publication. To support this, agreed funders will pay the cost of article publishing charges (APCs) (up to a yet unannounced limit) to journals that are immediately and wholly open access (sometimes referred to as ‘gold’ open access).

Whether health research funding bodies, prior to Plan S, provide funded researchers with guidance or support towards selecting publishing journals in line with their policies and which facilitate proper (and permanent) access to research, and whether they monitor such policies, is unknown. Previous studies confirm that many non-commercial health research funders’ have policies requiring open access to completed research or results via publication or otherwise ^33, 34^. Yet none seem to have assessed whether funders provide any specific information to researchers to facilitate their choice of publishing journal. For public or charitable funders, providing such guidance or support may be one way of ensuring responsible stewardship of public or donor funds. While research publication routes exist beyond scientific journals (e.g., preprint servers, repositories) the present project examines journals as the primary vehicles of research dissemination due to funders’ and academia’s reliance on them as a gauge of research impact/productivity. The current work will establish a pre-Plan S baseline of health research funders’ guidance on selecting journals in which to publish funded research.

The aim of this research is to describe the policies and recommendations of major health research funding bodies regarding suitable journals for funded research.

## Methods

We considered the public websites of 50 health funding bodies for statements, guidance, or policies specifically mentioning the publication of funded research. Detailed methods and rationale for this study are elaborated in an *a priori* study protocol ( https://doi.org/10.17605/OSF.IO/J6CSK) and summarized below.

### Data source

Global funding bodies with the largest documented health research expenditures were sampled from the curated Health Research Funder’s list found at: www.healthresearchfunders.org ^35^. The list was developed as part of an unfunded post-doctoral project by researchers in the Netherlands ^35^. It was last updated in 2016; expenditure data are reported in 2013 US dollars (USD, accounting for inflation/deflation rates by country). A detailed account of the systematic process used to identify funders and obtain expenditure data is found here: http://www.healthresearchfunders.org/faq/. At the time of retrieval for this study (August 2017), 287 health research funding bodies from 30 countries were included on the list. The list distinguishes five categories of funders: [1] philanthropic funders (n=194), [2] public funders (n=77), [3] public funders who fund health research through Official Development Assistance (public ODA) ^1^ (n=8), [4] multilateral funders (funding across countries) (n=7), and [5] public-private partnerships (PPP) (n=1) ( Table 1). While there are some inequities in its coverage (e.g. public funders were selected from only G20 countries; paucity of funders from low income countries), the list is likely the most comprehensive source of global health research funder expenditure information in existence (personal communication, Dr. Beverley Holmes, CEO, Michael Smith Foundation for Health Research) and has been used to construct samples in at least two other studies ^34, 36^. This study excludes commercial funders since their expenditure data are not publicly or readily available.

**Table 1.** Annual Expenditure across health research funders (in 2013 USD millions) by World Bank income level and type of funder. ^*^

World Bank Income Level 2014	Type of Funder annual expenditure in 2013 USD millions (# of funders) [range]				
	Philanthropic	Public	Public-ODA	Multilateral	PPP
**high income: non-OECD ^†^**	None listed	274.31(5) ^‡^	None listed	None listed	None listed
**high income: OECD**	4995.25 (194) ^§^	39847.47 (50) ^**^	344.40 (8)	None listed	None listed
**upper middle income**	No data (1)	1540.87 (13) ^††^	None listed	None listed	None listed
**lower middle income**	None listed	140.26 (4) ‡	None listed	None listed	None listed
**low income**	None listed	None listed	None listed	None listed	None listed
**Income level not stated**	None listed	6111.78 (5)	None listed	137.09 (7) ^**^	455.36 (1)

^*^Created from data at healthresearchfunders.org.

^†^ OECD: Organisation for Economic Co-operation and Development.

^‡^ Includes 3 funders with no expenditure data available.

^§^ Includes 11 funders with no expenditure data available.

^**^ Includes 1 funder with no expenditure data available.

^††^ Includes 5 funders with no expenditure data available.

### Sampling

To construct our sample, we sought up to 15 funders with the largest expenditures from each of the five funder categories from the list at www.healthresearchfunders.org, and aimed to include all listed lower income countries (n=4) if they were not otherwise represented in the sample. We included the latter group of funders in order to ensure representation from lower income countries, since researchers and journals from these countries have been disproportionately implicated in predatory publishing ^26, 37^. Working with the available number of funders in each category ( Table 1), we ended up with 50 funders: 15 philanthropic, 15 public, eight public ODA, seven multilateral, one PPP, and four lower-middle income country funders.

**Figure 1.**
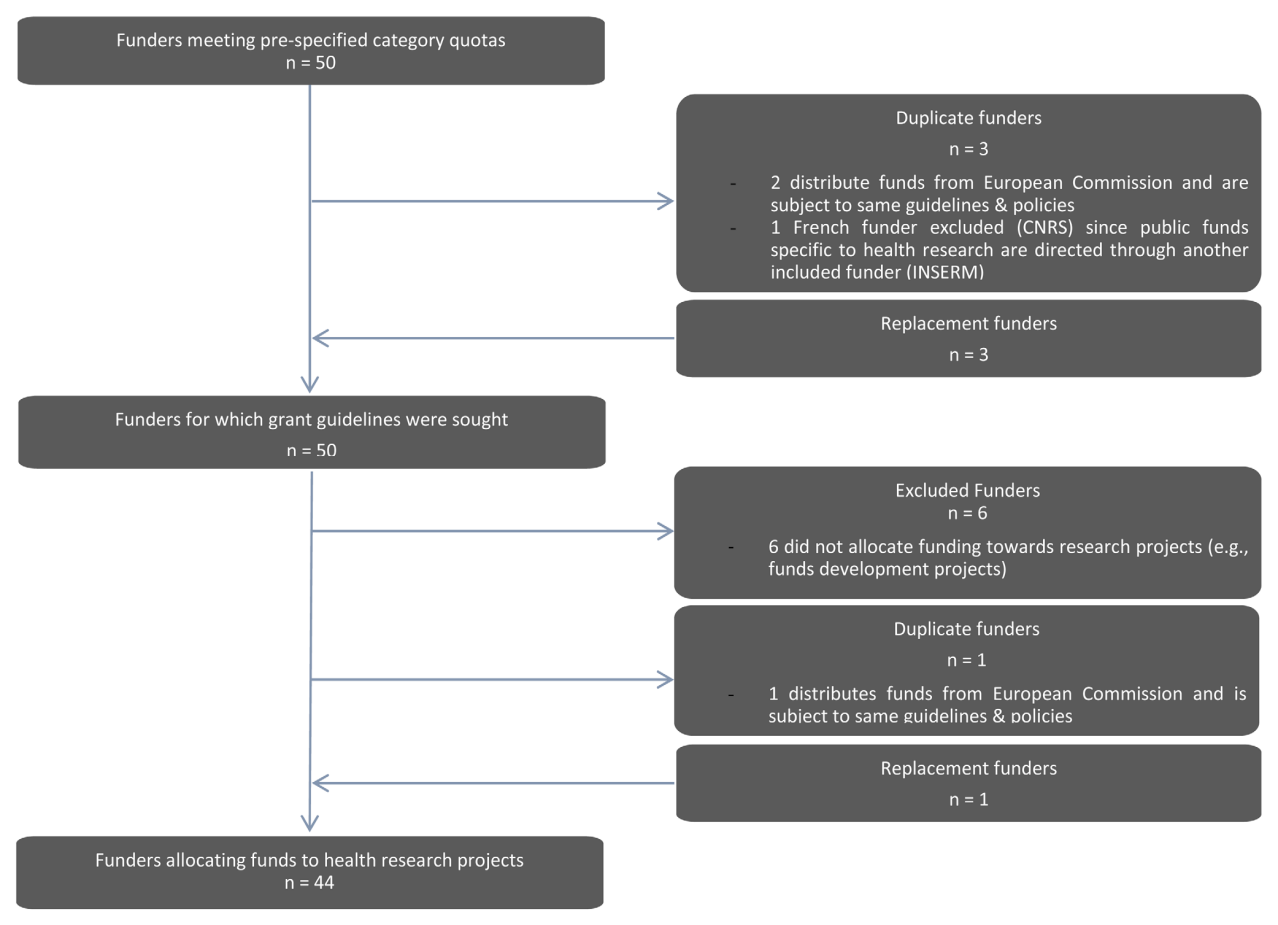
Flow diagram for selection of included funders.

In line with previous investigations into health research funder policies ^34, 38^, we expected that guidance for funded researchers would be publicly available and easily obtained. For each included funder, one of us (LS) visited the website using the URL provided by www.healthresearchfunders.org, or if the URL was not working, found it through a Google search using the funder name. When a funder’s website could not be located/did not work or when the funder was a duplicate, the next largest funder on the list was used. For each funder, we sought and downloaded the website section on policies for funded research in August-September 2017. If no specific policies were found, we searched the SHERPA (Securing a Hybrid Environment for Research Preservation and Access)/Juliet database ( www.sherpa.ac.uk/juliet/index.php), which lists and links conditions for open access publication for some funders (though this is incomplete as it is reliant on voluntary contributions from funders and other organizations [e.g., libraries] tracking funder policies). If a funder’s website did not mention funding health research (i.e., funded other scientific research) or if the funder did not specifically award grants for research, the funding body was excluded from the sample and replaced with the next largest funder (by expenditure), where possible. Reasons for exclusion are documented in Figure 1.

### Data extraction

One assessor (LS) extracted information from the downloaded policy documents into an online form in Distiller SR, and a second assessor (KDC or MJP) verified the extracted data. Discrepancies were resolved by discussion. If additional documents were identified during extraction, we saved them and searched them for the desired data. The verification process led to clarifications in collected data or provided additional information. Since no reference standard for funder policies on publishing exists, the extracted items were derived de novo by the study team; no formal consensus process was used. The following information was assessed or extracted, as available:

- Any statement(s) about the dissemination of outputs from funded research
- Policy or recommendations about publication of funded research
- Policies or recommendations on research/data accessibility
- Policies or recommendations on journal quality, impact factor or other metric, ethical standards, and indexing of funded research;
- Whether/what information is provided to researchers about predatory or substandard journals, or about journal credibility
- Strength of any aforementioned policies/recommendations (‘must’, ‘should’, or ‘suggested’)
- For publication policies, whether adherence will be monitored
- For publication policies, whether consequences of non-adherence are listed

If non-English websites or documents were encountered and an English-language version was not available on the website, Google Translate was used to automatically translate the websites and documents. Google Translate has recently shown 85%–97% accuracy across nine languages for translating health research studies ^39^, including the languages encountered in this study (French and German).

### Data analysis

We summarized data descriptively by calculating proportions for dichotomous data; the date of funder policies/recommendations were summarized as medians and interquartile range.

### Protocol deviations

In the protocol for this study, we stated that we wanted to determine whether there were differences in the number of funders with policies/statements about journal quality and predatory publishing based on the income-level of the funder country or country being funded. However, as only four funders from lower-middle income countries and none from low income countries were on the list we sampled from, there were not enough funders to enable meaningful comparisons between higher income and lower income countries.

## Results

For the 50 funding bodies originally identified using the described sampling technique, three allocated money from a funder (European Commission ^2^) already in the sample and were replaced with the next organizations on the list. One of the replacement funders also allocated money from an included funder and was also replaced. Two funders funded non-health research and four funders did not list any research grants (and appeared to fund health development initiatives) and could not be evaluated for our purposes. Overall, six funders were excluded and lacked replacements in the categories they belonged to. 44 funders remained in the sample for which grant policies and guidelines were sought ( Figure 1). 35 funders are from high income countries, one from upper-middle income (China), three are from a lower-middle income country (India), and five are not classified by income level because they are multilateral (n=3) or fund across the European Union (EU) (n=2, Table 2).

**Table 2.** Description of funders (n=44).

Descriptor		
Type of funder, n(%)	Philanthropic Public Public ODA Multilateral Public Private Partnership	15 (34%) 18 (41%) 7 (16%) 3 (7%) 1 (2%)
Country Income Level, n(%)	High income Upper-middle ^i^ Lower-middle Low income ^ii^ N/A ^iii^	35 (80%) 1 (2%) 3 (7%) 0 (0%) ^iv^ 5 (11%)
Annual expenditures (in million 2013 USD), mean (n)	High income Upper-middle Lower-middle Low income ^iv^ N/A ^iii^	$1,113.191 (35) $621.273 (1) $140.261 (1) - $862.024 (5)

^i^ China.

^ii^ All funders from India.

^iii^ Income-level not available since funders distributing funds across multiple countries (3 multilateral funders and 2 European Union funders).

^iv^ No funders from “Low income” countries in sample.

38 of 44 funders (86%) had publicly available information for grantees about disseminating funded research outputs ( Table 3). Of the six funders that did not have publicly available information, five are from high-income countries (US, Germany, France, UK) and one funds research in the EU through public-private partnership. Three are philanthropic organizations and two are public-ODA funders. Information about disseminating research was contained within “policies” for 29/38 (76%) funders, “recommendations” (suggestions and guidance) for 8/38 (21%) of funders, and as a “code of conduct” for one funder ( Table 3). All but one policy/recommendation referred to funded research (including results) as the unit of dissemination (37/38, 97%). Over a third of policies/recommendations also specifically mentioned the dissemination of “data” (25/38, 66%). The median implementation date or date listed on collected documents was September 2014 (IQR: Apr 2012 to Apr 2016, n=35).

**Table 3.** Funder Policies on publishing.

		n(%)
Publicly available webpage or document(s) on funder website discussing dissemination o research outputs ^a^		38 (86%)
Type of statement about research outputs ^b, c^	Policy Recommendation/ Guideline Other	29 (76%) 8 (21%) 1 (3%) ^d^
Dissemination pertains to specific research output ^b^	Research Data Other materials ^e^	37 (97%) 25 (66%) 14 (39%)
Date of effect ^f^ [median (IQR)]		Sept 2014 (Apr 2012– Apr 2016)

^a^ Denominator = 44 funders with grant guidelines.

^b^ Denominator = 38 funders with statements about research outputs.

^c^ Policy: uses the words “policy”, “must”, “require”; Recommendation/Guideline: uses the words “recommendation” “recommend”, “suggest”, “should”, “guideline”.

^d^ Other - described as “Code of Conduct”.

^e^ Verbatim: activities of funded organizations; all research outputs, news releases; photos; any and all other published material referencing the research project or grant; code; research materials; protocols; research resources including, but are not limited to, the full range of tools that scientists and technicians use in the laboratory, such as cell lines, antibodies, reagents, animal models, growth factors, combinatorial chemistry, DNA libraries, clones and cloning tools (such as PCR), methods, laboratory equipment and machines; presentations; media interviews; and other professional activities; 'research tools'; metadata; bibliographic metadata; supplementary materials; other supporting artefacts, research resources/tools.

^f^ Out of 35 funders listing this information. Date of implementation was used if available, otherwise, date of document or last update was used. When only year was given, January was used as default month; when a date range was given the most recent date was used.

### Open access and journal selection

36 of 38 policies/recommendations (95%) specifically referred to publication in a journal as one form of dissemination for completed research ( Table 4). 31 of these (86%) mentioned that research should be open access, either through journal publication (n=24, 77%) or through self-archiving the final report or accepted manuscript in a freely accessible repository (such as PMC) (n=30, 97%). One funder from India (Indian Council of Medical Research), one from France (Institut National de la Santé Et de la Recherche Médicale, INSERM), and three from the USA (US Department of Defense, Congressionally Directed Medical Research Program, and the American Cancer Society) did not mention open access in their policies about research dissemination.

**Table 4.** Funder position and information provided about journal publication of funded research (n=38).

		n (%)
Mentions journal publication of research outputs	Yes Mandatory Suggested/encouraged Can’t tell No (but sharing o research mentioned)	36 (95%) 0 36 0 2 (5%)
Offers guidance on journal selection ^1^	Yes No	13 (36%) ^2^ 23 (64%)
Mention of journals’ or publishers’ integrity or credibility. ^1^	Yes No	6 (17%) ^3^ 30 (83%)
Journal practices or characteristics mentioned ^1^	Open Access Journal Impact Factor (JIF) Any measure/description of journal quality ^4^ Peer review Any transparency or ethics standards Database indexing (excluding PMC) Other Nothing specific about journals mentioned	31 (86%) 0 4 (11%) ^5^ 30 (83%) 11 (31%) ^6^ 0 2 (6%) ^7^ 4 (11%)

^1^ Denominator: 36 funders mentioning journal publication.

^2^ See Table 5 for verbatim text of statements about journal selection.

^3^ See Table 5 for verbatim text of statements about journal credibility.

^4^ excluding JIF.

^5^ 2 funders indicate journal should be “high quality, peer reviewed journal”; 1 funder indicates journal should be “quality peer-reviewed journal”; 1 funder indicates what a good journal is: "Good journals have guidelines for reviewers of manuscripts committing them to strict confidentiality/to disclose conflicts of interest and promise to respond to submitted manuscripts within a specified, short time limit, and correspondingly set their reviewers short time limits for their comments."

^6^ See Table 6 for transparency or ethics standards for publications.

^7^ Other: 1 funder encourages publication in “primary scientific journals”; 1 funder states “models and mechanisms for publication and access to research must be both efficient and cost effective”;.

13 of 36 (36%) policies recommending publication contained some guidance on how to select a journal and six (17%) listed features or requirements of publishers or journals for researchers to look for ( Table 5). These six are described here. Only one funder policy (NIH) included a definition of a journal (i.e., either a publication listed in the journal section of the National Library of Medicine or one meeting stated criteria). And only one funder policy (Canadian International Development Research Council, IDRC) appeared to provide any information about ‘questionable’ journals in a guidance document entitled “Publishing in Open Access Journals”. The document lists journal features to “look for” and to “be wary of” and mentions Beall’s List ^3^ as a resource ( Table 5). One policy (Deutsche Forschungsgemeinschaft/German Research Foundation, DFG) linked to Think, Check, Submit ( www.thinkchecksubmit.org) – an initiative to facilitate researchers’ assessment of the credentials of a journal – on a page supplementing their open access policy listing open access resources. Two funders distributing APC fees through the Charitable Open Access Fund (Cancer Research UK and Wellcome Trust) list the requirements of journals whose APCs are eligible for payment through the fund. The Bill and Melinda Gates Foundation provide researchers with a portal (called Chronos) through which to submit manuscripts directly to pre-selected journals whose standards are in line with their requirements.

**Table 5.** Funder statements on journal selection, requirements & credibility.

Funder	Journal Selection	Specific publisher/journal requirements or acceptability/credibility features	Name of webpage/source document
**Bill & Melinda Gates Foundation**	“Open access publishing is a non-negotiable term included in all grant agreements as of January 1, 2015. Please log into Chronos to search and find journals that offer open access options as required by the foundation's policy.” “Our Open Access policy contains the following elements: 1. Publications Are Discoverable and Accessible Online. Publications will be deposited in a specified repository(s) with proper tagging of metadata. 2. Publication Will Be On “Open Access” Terms. All publications shall be published under the Creative Commons Attribution 4.0 Generic License (CC BY 4.0) or an equivalent license. This will permit all users of the publication to copy and redistribute the material in any medium or format and transform and build upon the material, including for any purpose (including commercial) without further permission or fees being required. 3. Foundation Will Pay Necessary Fees. The foundation would pay reasonable fees required by a publisher to effect publication on these terms. 4. Publications Will Be Accessible and Open Immediately. All publications shall be available immediately upon their publication, without any embargo period. An embargo period is the period during which the publisher will require a subscription or the payment of a fee to gain access to the publication. We are, however, providing a transition period of up to two years from the effective date of the policy (or until January 1, 2017). During the transition period, the foundation will allow publications in journals that provide up to a 12-month embargo period...”	“Chronos will list the most current information about which journals offer these [open access] options, i.e., the listing for Science will reflect the current agreement between the Gates Foundation and the American Association for the Advancement of Science (AAAS).”	Open Access Policy - Frequently Asked Questions
**Bloodwise**	“If the journal is not compliant with our open access policy, grant holders can request from Bloodwise that grant underspend is used to cover the cost with a justification for publishing in a non-compliant journal”/”Researchers can determine which publishers are compliant with this policy by referring to the SHERPA/FACT database and checking against the Wellcome Trust’s policy, which has the same stipulations.”	None stated	Project grants – guidance for applicants
**British Heart Foundation**	All of our Grantholders submitting manuscripts to journals should find out in advance whether the publisher supports open access and how they can comply with paragraph 2 above.	None stated	Open Access Policies
**Cancer Research UK**	"The journal you publish in must be published by a publisher who has agreed to the COAF/Wellcome Trust publisher requirements. Cancer Research UK and the Charity Open Access Fund (COAF) will only fund APCs where the publisher has agreed to the COAF/Wellcome Trust publisher requirements. These publisher requirements set out the open access services that publishers must agree to provide when they charge APCs for COAF-funded researchers. Most major publishers and many smaller publishers have signed up to these requirements. If you would like to check that the publisher of your chosen journal is compliant, search the SHERPA/FACT database here. Select the name of the relevant journal and Cancer Research UK as your funder – as long as the results show at least one ‘tick’ (✓), the journal is compliant by that route.”	“Publishers who want to provide open access publishing services for Wellcome and Charity Open Access Fund (COAF) grantholders through peer-reviewed journals based on article processing charges (APCs) must commit to providing a service that meets our requirements. Our open access policy (/funding/managing-grant/open-access-policy) states that when Wellcome funds are used to pay an APC, the article must be deposited, at the time of publication in PubMed Central (PMC). It will then be mirrored to Europe PMC. All deposited articles must be made available under the Creative Commons Attribution (CC-BY) Licence. COAF follows the same policy. Wellcome and COAF’s open access policies and requirements are designed to maximise the availability and reuse of publications. We hope that publishers will continue to support us and our grantholders in this endeavour. We recognise that relationships in the publication process are not straightforward – we have grant- funding relationships with researchers, who then establish relationships with publishers. This can lead to confusion about what Wellcome and COAF expect from publishers. Our requirements clarify the service that publishers who receive Wellcome or COAF funds need to provide.”	Open Access Policy/Publisher Requirements
**Deutsche** **Forschungsgemeinschaft** **/German Research Foundation**	“The approved funds are only for the publication of essays in original Open Access journals. The funds may only be spent provided that the following conditions are met:…The articles to be published appear in journals whose contributions are all accessible to users free of charge ("genuine open access journals") immediately after their publication on the Internet, and which apply the strict quality assurance procedures recognized in the relevant subject… The funds provided by the DFG may not be used to publish open essays in journals subject to subscription (according to the "Open Choice" model etc.) for Open Access.” * "Guiding ideas for choosing appropriate means of publication: http://thinkchecksubmit.org"	“Recommendation 12: Scientific journals shall make it clear in their guidelines for authors that they are committed to best international practice with regard to the originality of submitted papers and the criteria for authorship."/ "all good scientific journals report when a manuscript has been received and when – usually following peer review – it has been accepted"	Further information on Open Access
**German Federal Ministry of** **Education and Research (BMBF)**	"Scientific articles from projects funded by the BMBF should either be published directly under an open access model or be put into a suitable document server after expiry of an embargo deadline. The researchers remain free in their choice, whether and in which journal they want to publish"	None stated	Press Release announcing Open Access Strategy
**Howard Hughes Medical Institute**	"HHMI strongly encourages all HHMI laboratory heads to publish their original, peer-reviewed research in journals that make publications freely available and downloadable on-line immediately after publication (i.e. open access journals). If a laboratory head chooses to publish an original, peer- reviewed research publication on which he or she is a major author in a journal that is not open access, the laboratory head is responsible for ensuring that the publication is freely available and downloadable on-line as soon as reasonably possible after publication, and in any event within twelve months of publication."	None stated	Public Access to Publications
**Indian Department of** **Biotechnology (DBT)**	“The DBT and DST recognize the right of researchers to publish their work in journals of their choice, because researchers are the best judges of where to publish their work. The DBT and DST expect that the recipients of funding will publish their research in high quality, peer- reviewed journals.” AND “Results should be published in an appropriate form, usually as papers in refereed journals, with copies being made available through PubMed Central (PMC) and UK PubMed Central (UKPMC) as soon as possible and in any event within six months of the journal publisher’s official date of final publication.”	None stated	DBT and DST Open Access Policy
**Indian Department of Science &** **Technology (DST)**	*same as Indian Department of Biotechnology (DBT)*	None stated	DBT and DST Open Access Policy
**IDRC/Global Affairs Canada**	“Authors should carefully consider the quality standards of publications and ensure that appropriate peer review mechanisms are followed. The Directory of Open Access Journals, maintained by Infrastructure Services for Open Access (IS4OA), lists open access journals that have been reviewed for quality. Authors can also refer to the information guide Publishing in Open Access Journals, which lists journal quality indicators to evaluate potential publication outlets.”	While most open access journals are peer-reviewed and high quality, there are a number of questionable journals, i.e. journals that do not subscribe to most or any of the practices of legitimate, academic journals. There is no single rule or test to indicate whether an open access journal is reputable. But here is what you should look for and what you should be wary of to avoid publishing in a questionable journal: Look for ... • Journal scope is well-defined and clearly stated • Journal is affiliated with or sponsored by an established scholarly society or academic institution • Editor, editorial board are recognized experts in the field • Articles are within the scope of the journal and meet the standards of the discipline • Any publishing fees or charges are easily found on the journal website and clearly explained • Rights for use and re-use of content at article level (e.g., Creative Commons CCBY licence) are clearly indicated • Articles have DOIs (Digital Object Identifier, e.g., doi:10.1111/j.1742-9544.2011.00054.x) • An ISSN (International Standard Serial Number e.g. 1234-5678) has been assigned • Publisher is a member of Open Access Scholarly Publishers Association (OASPA) • Journal is listed in the Directory of Open Access Journals (DOAJ) • Journal is included in established subject databases and/or indexes (e.g., Academic Search Complete, Medline, etc.) Be wary of ... • No journal scope statement or one that is vague • Website mimics other well-known publishers’ site, or links or uses logos of recognisable entities although there is no actual connection • Journal title is very similar to title of a more established journal • Poorly maintained Web presence, including dead links, multiple spelling and grammatical mis- takes (on website and in articles) • No editor, editorial board, or editorial staff is listed, or they lack affiliation • Publisher is also the editor of the journal and/or editorial boards members serve on the board of multiple titles from the same publisher • Authors have several articles in the same issue • Publisher uses direct and unsolicited marketing (i.e., spamming) or advertising is obtrusive (to publish articles or serve on editorial board) • No information is provided about the publisher or location; either information is missing or does) not match geographical area covered by the journal (e.g., “American Journal of ….” but published in) Croatia) • Instructions to authors regarding peer review, copyright and/or fees (APCs), are not listed on web-site or are unclear • Publisher promises unusually rapid peer review and publication schedule Open Access • New (but self-proclaimed “leading”) publisher with a large number of journals • Journal not listed in the Directory of Open Access Journals (DOAJ) • Claims to be indexed by Google and Google Scholar (or other search engines that crawl the Web) • Refers to bogus metrics meant to mimic Impact Factor (e.g., Journal Influence Factor, Global Impact Factor, etc.) • Publisher has a negative reputation (e.g., documented examples in Chronicle of Higher Education, list-servs, etc.) • Journal or publisher appears on Beall’s List of Predatory Journals and Publishers Tools to find and evaluate open access journals: • Directory of Open Access Journals: http://doaj.org/ Online index of open access, peer-reviewed journals • Beall’s List of Predatory Journals and Publishers: http://scholarlyoa.com/ List of potential, possible or probable questionable scholarly open access publishers and standalone journals • SHERPA/RoMEO: http://www.sherpa.ac.uk/romeo/ Online index of publisher copyright and self-archiving policies	Open Access Policy for IDRC- Funded Project Outputs/IDRC Publishing in Open Access Journals
**National Health & Medical** **Research Council (Australia)**	"NHMRC acknowledges that researchers take into account a wide range of factors in deciding on the best outlets for publications arising from their research. Such considerations include the status and reputation of a journal, book, publisher or conference, the peer review process of evaluating their research outputs, access by other stakeholders to their work, the likely impact of their work on users of research and the further dissemination and production of knowledge. Taking heed of these considerations, NHMRC wants to ensure the widest possible dissemination of the research supported by NHMRC funding, in the most effective manner and at the earliest opportunity.	None stated	NHMRC OA policy
**National Institutes of Health**	“Awardees must ensure that any publishing agreement allows the paper to be posted to PMC in accordance with the NIH Public Access Policy. NIH does not dictate the means by which awardees must do so, but does offer guidance in an FAQ on suggested wording for publishing agreements.”	"If a publication is in the journal section of the NLM catalog, NIH considers it to be a journal. Search the journal section of NLM Catalog ( http://www.ncbi.nlm.nih.gov/nlmcatalog/journals) for the journal by title, title abbreviation, or ISSN. Automatic suggestions will display as you type. If the publication is not on the list, NIH will consider it a journal for policy purposes if it meets all of the following criteria: Publication must meet the requirements for ISSN assignment; Publication content is issued over time under a common title; Publication is a collection of articles by different authors; Publication is intended to be published indefinitely; You may also submit the manuscript to NIHMS upon acceptance for publication for a determination."	National Institutes of Health Plan for Increasing Access to Scientific Publications and Digital Scientific Data from NIH Funded Scientific Research/NIH FAQ on Public Access Policy
**Wellcome Trust**	“We expect Wellcome-funded researchers to select publishing routes that ensure the work is available immediately on publication in its final published form, wherever such options exist for their publisher of choice and are compliant with our policy.” “The [Charitable Open Access Fund] fund can only be used for APCs where the publisher has agreed to our publishing requirements.” “Check that your journal of choice has a publishing policy compliant with our grant conditions. Wellcome-funded authors can use the SHERPA Funders’ and Authors’ Compliance Tool ( http://www.sherpa.ac.uk/fact/) (SHERPA FACT) to check this.”	*Same as Cancer Research UK*	Open Access Policy/Publisher Requirements

^*^ “Open Choice” is a term used by the publisher *Springer* to refer to hybrid journals.

**Table 6.** Transparency tools/activities mentioned by funders.

Funder	Additional publication transparency actions/initiatives mentioned	Source
**Bill & Melinda Gates** **Foundation**	“Publications Are Discoverable and Accessible Online: Publications will be deposited in a specified repository(s) with proper tagging of metadata.”	Open Access Policies
**Bloodwise (formerly** **Leukaemia & Lymphoma** **Research)**	"Grant holders should make use of the ARRIVE guidelines when designing their experiments, and ensure that they report in vivo studies in accordance with the ARRIVE guidelines as far as possible."	Project Grants Guidance
**Deutsche** **Forschungsgemeinschaft /** **German Research Foundation**	Recommendation 7: Safeguarding and Storing of Primary Data. "Experiments and numerical calculations can only be repeated if all important steps are reproducible. For this purpose, they must be recorded. Every publication based on experiments or numerical simulations includes an obligatory chapter on “materials and methods” summing up these records in such a way that the work may be reproduced in another laboratory."	Safeguarding Good Scientific Practice
**European Commission**	“To be able to easily find the deposited publication, beneficiaries must also ensure open access – via the repository – to the bibliographic metadata that identify the deposited publication. This metadata must include a persistent identifier (such as the Digital Object Identifier, DOI) in order to allow easy and persistent referencing.”	European Research Council (ERC) Guidelines on Implementation of Open Access to Scientific Publications and Research Data
**Indian Council of Medical** **Research**	Researcher’s Relations with the Media and Publication Practices: “The term ‘misconduct in research’ means fabrication, falsification, plagiarism, selective omission of data and claiming that some data are missing, ignoring outliers without declaring it, not reporting data on side effects/ adverse reactions in a clinical trial, publication of post-hoc analysis without declaring it, gift authorship, not citing others’ work, not disclosing conflict of interest, redundant publication, and failure to adequately review existing research. The Commission on Research Integrity in US created by US Congress addresses the scientific, ethical, social and legal issues involving scientific misconduct in research. Consolidated standards of reporting trials (CONSORT) guidelines have been prescribed for publishing results of clinical research especially RCTs (Randomised Controlled Trials) and are available at http://www.consort-statement.org/."	Ethical Guidelines for Biomedical Research on Human Participants
**Institut Pasteur**	“To be able to easily find the deposited publication, beneficiaries must also ensure open access – via the repository – to the bibliographic metadata that identify the deposited publication. This metadata must include a persistent identifier (such as the Digital Object Identifier, DOI) in order to allow easy and persistent referencing.”	European Research Council (ERC) Guidelines on Implementation of Open Access to Scientific Publications and Research Data
**Medical Research Council (UK** **Research and Innovation since** **2018)**	G.8. Reporting Guidelines: "Agreed standards for reporting the outcomes of research in specific areas have been developed and should be observed. Standards endorsed and supported by the MRC include the CONSORT Statement (CONsolidated Standards of Reporting Trials) ^40^ and the ARRIVE guidelines (Animal Research: Reporting in-vivo experiments) ^41^."	MRC ethics series. Good research practice: Principles and guidelines
**NIH**	5.a. Access & Discoverability: Meta Data. “Ensure full public access to publications’ metadata without charge upon first publication in a data format that ensures interoperability with current and future search technology. Where possible, the metadata should provide a link to the location where the full text and associated supplemental materials will be made available after the embargo period; Ensure that attribution to authors, journals, and original publishers is maintained (OSTP memo, elements 3c, 3e)”	Plan for Increasing Access to Scientific Publications and Digital Scientific Data from NIH Funded Scientific Research
**U.S. Agency for International** **Development**	“Metadata [from a research dataset or publication] should be made accessible as soon as possible after final acceptance of a paper and appropriate review, even if the full text is subject to an embargo period.”	Public Access Plan
**World Bank**	“Requires electronic copies of complete, final manuscripts, as defined above, as well as the associated metadata, to be deposited in the Open Knowledge Repository”	World Bank Open Access Policy for Formal Publications
**World Health Organization**	Summary of the open-access policy options and requirements: “Publisher deposits XML version of the article and metadata in PubMed Central, which automatically appear in Europe PubMed Central”	WHO open-access policy: Frequently asked questions for recipients of WHO funding

The policies of at least three funders (German Federal Ministry of Education and Research Indian [BMBF], Indian Department of Biotechnology [DBT], Indian Department of Science & Technology [DST]) include a statement that further to making research freely accessible, researchers’ choice of journal was unrestricted.

### Other journal characteristics mentioned by funders

Most funders mentioned that funded research should be peer reviewed or published in a peer reviewed journal ( Table 4). Four funders made non-specific reference to selecting a “good” or “quality” journal in relation to publication of funded research; none mentioned the journal impact factor. Eight funders made statements about publication transparency or ethics. For instance, one funder discussed reproducibility in published research, three mentioned adherence to reporting guidelines, and at least six asked that metadata accompany published articles ( Table 6).

### Adherence to policies/recommendations

Of 38 policies/recommendations providing information about disseminating research outputs, only nine (24%) stated that they monitor adherence to either a policy (n=7) or recommendation (n=2); two philanthropic funders (Wellcome Trust and Bill & Melinda Gates Foundation) specified that they would evaluate publications of funded research reported to them to ensure they are published in journals meeting the funder’s outlined publishing requirements ( Table 7). No monitoring or adherence data appears to be publicly available. Only five (13%) funders with policies or recommendations about journal publication indicated that there would be consequences for non-adherence. And only two of those (Wellcome Trust and NIH) stated that they would withhold or suspend payments if articles are not made open access.

**Table 7.** Monitoring and consequences.

Funder Name	Adherence-monitoring strategy	Consequences for non-adherence
**American Heart** **Association**	(From OA policy) "In situations where the AHA or members of the research community feel that AHA-funded researchers are not sharing data in a manner consistent with our policy, you may be asked by the AHA to demonstrate your compliance.”	“If you cannot do so [demonstrate compliance with OA policy], this may affect future funding.”
**Bill & Melinda** **Gates**	(From FAQ about OA Policy) "The foundation checks and tracks compliancy through Chronos, a new service to help you manage the process of publishing under the policy’s terms."	*None stated/identified*
**Canadian** **Institutes for** **Health Research**	*Nothing stated/identified*	*not specific to journal publication - but have consequences for breaches in policy,* *including breaches around dissemination of research*
**Centers for** **Disease Control** **and Prevention**	(From…) “ Supervisors of publishing authors are responsible for ensuring compliance with the CDC Public Access Policy.”	*None stated/identified*
**Institut Pasteur**	(From OA intentions) “The deposit is checked by the CeRIS library's Hal team, in particular when it comes to the rights of authors on their files and their affiliations to a lab and and institution.”	*None stated/identified*
**JRDF**	*Nothing stated/identified*	(From Award Terms and Conditions) *not specific to publication* “JDRF may place the PI and/or his/her Grantee Institution on administrative probation if …the Grantee Institution is non-compliant as outlined in this document. JDRF administrative probation may include, but is not limited to, the following actions: 1. Withholding of all payments for the grant/project in question 2. Changing to a payment reimbursement schedule 3. Withholding of all JDRF payments for the PI, for any JDRF grant 4. Withholding of all JDRF payments fo the Grantee Institution, for any JDRF grant 5. Any combination of the above”
**National Institutes** **of Health**	(NIH Public Access Policy) “Compliance with this Policy remains a statutory requirement and a term and condition of the grant award and cooperative agreement, in accordance with the NIH Grants Policy Statement.”	(NIH Public Access Policy - FAQ) “A grantee’s failure to comply with the terms and conditions of award may cause NIH to take one or more enforcement actions, depending on the severity and duration of the non-compliance. NIH will undertake any such action in accordance with applicable statutes, regulations, and policies. NIH generally will afford the grantee an opportunity to correctvthe deficiencies before taking enforcement action unless public health or welfare concerns require immediate action. However, even if a grantee is taking corrective action, NIH may take proactive action to protect the Federal government’s interests, including placing special conditions on awards or precluding the grantee from obtaining future awards for a specified period, or may take action designed to prevent future non-compliance, such as closer monitoring. See Enforcement Actions in the NIH Grants Policy Statement (10/12)..”
**Pan American** **Health** **Organisation**	“Research commitments shall be reflected in institutional policies and program budgeting and planning, implementation, monitoring, and evaluation; human resource management; and knowledge management.”	*None stated/identified*
**UK Department** **for International** **Development**	(From DFID Research Open and Enhanced Access Policy) “As part of its monitoring and evaluation framework, DFID Research and Evidence Division (RED) collects data on the extent to which researchers fulfil the requirements and recommendations of this policy. RED researchers are required to report open access activity, usually during the annual review.”	*None stated/identified*
**US Agency** **for International** **Development**	(From Public Access Plan) “Potential future monitoring plans are suggested: "Further criteria/ metrics for compliance and evaluation will be developed through consultations with M/OAA/P, GC, and other USAID Operating Units. This may include: A) requesting offerors to submit within their proposals a list of USAID-funded peer-reviewed publications (and the corresponding DEC-issued unique identifier number for publication) that resulted from their prior USAID awards when deemed appropriate by the CO; B) importing the metadata and links to the authors’ manuscript or the publically available version of record of publications that resulted from USAID support, but were published after completion of the award in partnership with third-party reference services such as CHORUS and FundRef; and C) including compliance with open data and public access requirements of awards as a measure of past performance."	*None stated/identified*
**Wellcome Trust**	(From Complying with our OA policy) "We actively monitor research papers authored by our funded researchers to ensure they comply with our policy. This includes the review of publications listed in ongoing grant reporting and in end of grant >reports. In addition, Wellcome-funded research papers detailed in applications submitted to us are reviewed to ensure compliance."	(From Complying with our OA policy) "When Wellcome-funded researchers do not comply with our open access policy, we will apply these sanctions: Applicants will be required to ensure that all their Wellcome-funded original research papers resulting from current or previous grants are compliant before we issue formal notification of any funding renewals or new grants. Where non-compliant research papers, book chapters and scholarly monographs are identified in an end of grant report, we will withhold the final 10 per cent of the total grant budget until all outputs comply. See our 10 per cent retention policy."

## Discussion

Most health research funders appear to have active policies about the dissemination of funded research, typically about open access which often include statements about journal publication. Few policies contain guidance on how to select journals, list features of journals meeting funder requirements, or about the credibility of publishing outlets. Only one health research funding organization (IDRC) made specific reference to the “questionable journals” at the time of data collection (August-September 2017). Additionally, few policies describe whether funded outputs are monitored for compliance with funders’ dissemination policies, and few describe any consequences for researchers’ non-adherence to their policies. Information is not available on whether the NIH or Wellcome Trust, both of whom promise to withhold or suspend grant funds for breaching their open access policies, have actually ever done so ^9^.

For many of the funders in our sample, information to guide research publication was found across multiple documents and not always within open access policy statements/documents where publication is mentioned. For example, the only guidance we identified that referred to predatory journals (IDRC) was contained in a PDF (entitled “Publishing in Open Access Journals”) separate from the funders’ main open access policy. The policy did not flag that the document contained information about predatory/questionable/non-credible journals. This unobvious placement of guidance or expectations around journal selection relies on researchers’ curiosity or knowledge that important information may be located outside of the main policy webpages or documents. If funders wish to provide guidance about journal credibility and predatory publishing, they may reach more researchers (and increase the likelihood of them reading it) by including such information within their main policies.

### Comparison to other research

At least four previous studies examining health research funder policies on clinical trial transparency have collected information on funder’s recommendations for disseminating research. 

Two studies using similar methods evaluated trial transparency policies (i.e., those related to trial registration, access to summary results, individual data availability) for non-commercial health research funders globally (n=18) ^34^ and in the USA (n=9) ^36^. After accounting for three common funders across studies, 21 of 24 (87.5%) funders (16 of which are represented in our study) either required or supported publication or registration of trial results (neither study or their available data distinguished between publication or registration). This is in line with our findings in which 86% (38 of 44) funders had such policies/recommendations.

A third study, published in 2017 which examined research waste-reducing policies of 11 non-commercial funders (six of which are represented in our study) reported six to be explicit in requiring publication of full reports of funded research ^33^. In comparison, 36 of 38 policies/recommendations (95%) in our study referred to journal publication as one form of dissemination for completed research but did not indicate that it was mandatory. There may be differences in how authors of that study and interpreted language in documents or policies. The names of the six funders ‘requiring’ publication in that study were not obvious in either the publication or available data, so we are unable to investigate this further.

A study published in 2008 examined 73 UK and international non-commercial health research funders’ guidance for reporting funded clinical trials ^42^. 49 funders (67%) explicitly stated that trials could or should be published. Of the three funders appearing in the 2008 sample and ours, all have maintained recommending (but not requiring) the publication of trial results. Whether funders provided any guidance on selecting a journal to publish in was not collected in the study.

No previous studies appear to have investigated whether health research funders’ provide guidance to help funded researchers select a journal for publication. Our study appears to be the most comprehensive investigation on this matter. This is surprising since our findings suggest that funders in our sample regard publication as the primary means of disseminating funded research. Further, studies show that researchers view journal publication as the primary way of disseminating research ^43, 44^.

### Strengths and limitations

This study is the first to examine the information funders provide researchers about selecting a journal in which to publish funded research. All funders in our sample that mention journal publication or provide guidance on selecting journals, do so within their open access policies. In a time where the scholarly publishing landscape has been infiltrated and confused by predatory journals, inadvertently resulting in some researchers trying to achieve open access to publish in predatory journals ^45^, funders can play a critical role in steering researchers in the right direction. Funders can be specific and explicit with regards to which journal features researchers should look for in order to select one that meets their open access requirements.

This study provides a benchmark by which to monitor how major health research funders are performing pre and post Plan S implementation (January 2021). Data collection occurred in August & September 2017, prior to the September 2018 announcement of Plan S. So far, 24 funders have committed to implementing Plan S, five of which were considered in this research (European Commission, Gates Foundation, MRC/UK Research and Innovation, Wellcome Trust, and the World Health Organization). Two of these, the Wellcome Trust and Gates Foundation, provided guidance (in the form of tools) to facilitate selecting a journal in line with their open access policies at the time of sampling. At least one funder (Wellcome Trust) has made changes to their open access policies in anticipation of Plan S ^46^.

Our study relied on publicly available information about funder expectations of funded research and was abstracted by a single person with verification by a second (i.e., not two independent people). Six funders in our sample did not provide any relevant public information. We did not seek verification on policies from funders. Data were collected at a time when publishing activities, particularly open access, was rapidly changing, in part in response to funded research being published in predatory journals ^45, 47^. We are aware that the NIH issued a notice on their Public Access Policy in November 2017 (outside of our sampling and data collection period) with recommendations to publish funded research in journals with ‘credible practices’ ^48^. Engaging funders in our study may have had the added benefit of increasing uptake of our findings/recommendations into practice.

The focus of this research is limited to health research funders. We have not accounted for or evaluated other potential scientific publishing gatekeepers such as academic institutions, governments, or companies carrying out scholarly research, despite the important role they can play ^49^. 

### Implications and recommendations for funders

***Explicit funder policies on publication expectations.*** Selecting a journal in which to publish research is not a straightforward undertaking ^40^, particularly since the emergence of predatory journals. For funders looking to make their expectations around publishing funded research more explicit and more transparent, we propose several recommendations on how this might be achieved in Box 1, based on findings of this research and on the expertise of authors. Providing specific information about journal considerations in funders’ policies to funded researchers may facilitate more thoughtful and responsible selection of journals. Several recommendations in Box 1 pertain to the explicitness of article/journal considerations mentioned in Plan S (e.g., persistent identifiers for publications; long-term preservation/archiving; article-level metadata). All health research funders may wish to consider making aspects of their policies that pertain to publishing more explicit, whether or not they intend to implement Plan S.

**Box 1. Recommendations for providing explicit/transparent guidance on journal selection in health research funders' open access policies**

1. Use precise wording to describe your agency’s expectations that funded research be published

- Indicate whether researchers are expected to publish their research (e.g. use of “must” vs “should”)
- indicate whether open access publication is one of several options for meeting the agency’s open access requirements.

2. Provide a definition of a journal that is suitable to your agency

◦ Decide what essential features a publishing entity should and should not have in order to be considered a suitable place for publication.
◦ Consider referring to/including the Committee on Publication Ethics (COPE) list of Core Practices all journals and publishers should follow: https://publicationethics.org/core-practices
◦ The NIH definition of a journal is ^41^:
  • Publication meets the requirements for ISSN (International Standard Serial Number) assignment;
  • Publication content is issued over time under a common title;
  • Publication is a collection of articles by different authors;
  • Publication is intended to be published indefinitely.

3. Indicate your agency’s requirements for access and discoverability of published articles

◦ Distinguish between free vs open access:
  • Published articles are free to access; AND additionally, for open access,
  • Licensing for published articles permit reuse and building on (typically through a Creative Commons Attribution License, CC BY).
◦ Ensure that published research can be accessed in perpetuity
  ○ Researchers can determine whether the publishing journal has a permanent archival arrangement in place either through automatic deposition to PMC ( https://www.ncbi.nlm.nih.gov/pmc/journals/), or to another archive (via the Keepers Registry: https://keepers.issn.org/keepers-registry) ^xxv^
  ○ PMC-partnered funders can require that researchers upload published research directly to PMC
◦ Journal provides unique permanent identifiers (e.g. digital object identifier [DOI]) (can check if journal/publisher is registered with CrossRef: https://www.crossref.org/06members/51depositor.html)

4. Be clear about your agency’s support for article processing charges arising from publication of funded research

◦ Indicate how much money is available each open access publication (e.g. maximum APC amount)
◦ Indicate who will receive APC payment from the funder – the author (institution) or the journal
◦ Indicate when funding will be distributed to support article processing charges
◦ Indicate whether there are any conditions on distribution of APC funds

5. Indicate whether your agency requires archiving in a repository alongside publication

◦ Indicate whether the publication, data, or both, are expected to be deposited in a repository
◦ Indicate when deposition is expected to occur (i.e., immediately or within a specified time frame)
◦ Indicate whether you have a dedicated repository for research publications (e.g., PMC for NIH-funded research), and if not, suggest one or more repositories that are considered acceptable by your agency
◦ Be clear that it is the authors’ responsibility to ensure publications are deposited in a repository
◦ Provide instructions/link to resources on how to deposit research in the suggested repository.

6. Indicate how your agency will monitor that funded research is published in appropriate journals, in line with agency recommendations/mandates

◦ For ease of monitoring, Provide instructions for researchers about where and how to include the funding agency name and grant number in published articles (guidance here: https://www.ukri.org/wp-content/uploads/2020/10/RIN-251020-FundersAcknowledgementInScholarlyjournalArticles.pdf)
◦ Provide instructions on if, how, and when to submit publications of funded research to the funding agency, or state how publications will be monitored otherwise
◦ Provide specific actions/consequences that the agency will carry out when funded research is published in a journal that does not meet agency requirements

^xxv^provides global monitoring of archiving arrangements for electronic journals.

The NIH is the only funder in our sample to clearly describe what it considers a journal – either those listed in the journal section of the National Library of Medicine (NLM) ( https://www.ncbi.nlm.nih.gov/nlmcatalog/journals) or those meeting a comprehensive set of criteria ^41^: (1) meets the requirements for ISSN (International Standard Serial Number) assignment; (2) content is issued over time under a common title; (3) is a collection of articles by different authors; and (4) is intended to be published indefinitely. All but the final criterion are straightforward to judge; presumably it is meant to distinguish a journal from a book or a monograph however NIH or NLM do not provide guidance on how to judge this criterion. Whether and how we can predict journals’ intentions to publish indefinitely has not been described. A more meaningful criterion for distinguishing journals from non-journals may be whether the publishing entity has archival arrangements in place (e.g., with LOCKSS, Portico, PubMed Central) to ensure perpetual access to content in the event a journal ceases to operate. Since preserving publisher content may have associated costs ^50^, predatory or non-credible journals (which some describe as “primarily fee-collecting operations” ^51^) may be unlikely to seek this service.

We surprised that the three funders from India in our sample (Indian Council for Medical Research, DBT, and DST) did not mention journal credibility or predatory journals, and further, that a common policy for two Indian funders (DST and DBT), dated December 2014, recognizes “the right of researchers to publish their work in journals of their choice, because researchers are the best judges of where to publish their work”. Since at least 2016, there has been an ongoing national initiative combat predatory journals and to support researchers in their choice of journals across higher education institutes in India. The main product of this work has been a list of approved journals in which academic researchers are permitted to publish in as well as standard templates for researchers when communicating with journals ^52^. The University Grants Commission (UGC), the regulator and funder of high education, has been leading the initiative. It is uncertain whether the country’s largest health research funders are on board due to their lack of guidance in this space. A coordinated approach by a range of stakeholder groups ^49^, which includes funders (who have innate authority to implement mandates about publishing), may facilitate improved publication decisions by researchers. Importantly, however, UGC’s list of approved journals has been plagued with numerous credibility concerns in its short existence ^53, 54^. Explicit recommendations from India’s funders regarding credible and non-credible features of journals in which to publish may be warranted in the absence of a trusted and comprehensive list.

***Facilitating and monitoring adherence to funder policies.*** Funders are well-positioned to provide researchers with resources and tools to help ensure that results from funded research are published in credible and discoverable journals, in line with their policies. Several organizations in our sample consistently offer more information about potential publishing routes and tools to facilitate adherence to their policies. We provide a list of tools to facilitate the development of funder policies on research outputs, adherence to such policies, and monitoring of policy adherence ( Table 8).

**Table 8.** Resources and tools that funders can use to support development of, adherence to, and monitoring of open access publishing policies.

Resource/Tool	URL	Origin	Description	Relevance & Implementation	Limitations
CHORUS	https://www.chorusaccess.org/	Association of American Publishers	A service that utilizes metadata from CrossRef Funder Registry and partner publishers to monitor the # of published articles and % that are freely accessible; prompts free article access if required by funder policies; ensures digital preservation.	FACILITATING ADHERENCE & MONITORING: Encourage researchers to include funder name and grant number in published articles. Ensure funder information is registered and correct in CrossRef Funding Registry.	Fee-based Only able to facilitate open access & preservation for publisher partners (paid service).
Chronos	https://chronos-oa.com/	The Bill & Melinda Gates Foundation	A service facilitating funders’ management and tracking of the publication process for funded research outputs, through which pay APCs, and by which to manage subsequent archiving. Automatically collects information on where research was published, whether, when, and in which repositories published research and data were deposited, and the type of license research was published under.	FACILITATE ADHERNCE: Require that researchers use Chronos to submit any funder-supported research for publication; pay APCs directly to publishers instead of distributing as grant funding. MONITOR ADHERENCE: Use to monitor # of open access publications. Use as grant reporting tool to alleviate researchers of administrative responsibility.	Fee-based Only tracks publications submitted through Chronos.
CrossRef Funder Registry ^xxvi^	https://search.crossref.org/funding	CrossRef	Tracks any published articles with a digital object identifier (DOI) issued by Crossref (main DOI registration organization) and for which funder metadata is available	MONITOR ADHERENCE: Ensure funder information is registered and correct in the Registry; use to search for publications and other research outputs with DOIs funded by a specific grant or by a funder overall.	No distinction between freely available vs. subscription content. Only able to track articles for which publishers have registered DOIs and funding metadata with Crossref (paid service). Dependent on authors reporting funding information during the submission process or on publishers extracting from article.
HowOpenIsIt? Guide to Research Funder Policies	http://www.orfg.org/policy- development-guide/	Open Research Funders Group; SPARC	Freely available framework for designing funders’ open access policies. Provides policy examples along a spectrum of openness for five aspects of research outputs: article access, data and code access, reuse, costs, and compliance.	POLICY DEVELOPMENT: Use to facilitate design of a policy that includes recommendations on one or more aspects of openness of research outputs, that are feasible/desired.	None
OpenAire	https://monitor.openaire.eu/	European Commission/ Horizon 2020	Uses metadata provided by a funder (i.e., grant number) to search for funded research outputs deposited in digital archives/repositories.	MONITOR ADHERENCE: Use to collect data on research outputs (including publications) that are available in repositories.	Only sees freely available outputs. Dependent on researchers including grant numbers within research outputs deposited in repositories. Relies on repositories being compliant/ providing necessary metadata.
PubMed Central (PMC)	https://www.ncbi.nlm.nih.gov/pmc	NIH	The US National Library of Medicine’s (NLM) repository of freely available, full-text, published biomedical and life sciences articles; Dedicated repository for published NIH-funded research and for select other US and international funders. Publications can be deposited by journals with agreements with PMC or by researchers reporting NIH (or other agreed funder-) supported research.	FACILITATE ADHERNCE: Take appropriate steps to partner with PMC, Europe PMC, or the Health Research Alliance (NLM partner): https://www.ncbi.nlm.nih.gov/ pmc/about/public-access/. Require that all publications from funded research are deposited in PMC via a publishing journal or by authors.	Relies on journals agreeing to deposit to PMC or on authors to deposit published research for journals without PMC agreements.
SHERPA/FACT (Funder & Author Compliance Tool)	http://sherpa.ac.uk/fact/	Jisc; Wellcome Trust; Research Councils UK	Free online tool for researchers funded by the Wellcome Trust and members of Research Councils UK to check whether their intended journal meets funder open access requirements.	FACILITATE ADHERNCE: Request inclusion to be listed in FACT from Jisc. Direct funded researchers to use FACT to select a journal in which to publish in.	Relies on authors to use. Restricted to searching journals for which open access policies that have been collected
Think, Check, Submit	https://thinkchecksubmit.org	Publishing community led (collaboration among many publishing professionals/ organizations)	Freely available checklist to facilitate researchers in selecting a relevant and trustworthy journal in which to submit their research for publication consideration	FACILITATE ADHERNCE: Encourage authors to use checklist to facilitate selection of credible journal.	Relies on authors to read/understand/use Uncertain development process and validity of checklist items.

^xxvi^Previously known as FundRef (until Nov 2015).

Monitoring researchers’ adherence to their policies may help funders understand the extent to which researcher’s publishing practices are guided by their policies ^55^. Informing researchers that their adherence to open access policies is being monitored may facilitate better awareness of such policies and potentially increase adherence to them ^56^. A 2018 study examining the accessibility of research supported by 12 research funding agencies across North America and Europe with open access policies, found that 62% of almost 1.3 million articles over nine years were freely available ^9^. In 2016, 90% of published research supported by the NIH and Wellcome Trust was free to access (via journal, repository, or both) ^9^. Both agencies mandate the deposit of published research by publishing journals or funded authors into dedicated repositories (PMC for NIH; PMC Europe for Wellcome Trust). The remaining 10 funders in the sample did not mandate depositing in a repository alongside publication and had lower rates of freely accessible articles. For example, for the Canadian Institutes of Health Research (CIHR) only 55% of published research was freely accessible in 2016, even though the funder had a dedicated repository (PMC Canada) until 2018 (it closed due to low usage and high upkeep costs) ^57^. The study’s authors conclude that funders with low compliance rates used less enforcement and had less infrastructure to support compliance with their open access mandates ^9^.

### Areas of future research

An important area of future study is whether researchers are being funded on the basis of grant applications that include research published in predatory journals – or in journals that may not be indexed in trusted databases. Predatory journals have made their way into consideration (via CVs submitted by researchers or through institution-initiated database searches) into applications for academic career advancement ^58– 60^. Some have called for such publications to either be discounted from consideration or for researchers who submit them for consideration to be prevented from career advancement overall ^61, 62^. It is unknown whether researchers are including publications in predatory journals as part of their funding applications. This should be evaluated. If they are, funders may wish to consider whether this is an important consideration for awarding funding.

## Conclusion

Most large health research funders mandate open access to funded research outputs, typically by way of open access journal publication and by deposition of published research in digital repositories. Few funders provide guidance on what constitutes a journal (or an open access journal) or are checking to ensure that published research that they have funded is indeed meeting specified requirements about how research should be shared. Health research funding organizations have an obligation to support researchers in meeting their mandates so that research can, as intended, contribute to the broader evidence base. The publishing community needs to provide guidance to funders and researchers on universally acceptable and transparent standards for journal operations. Many solutions to improve policies, facilitate adherence, monitor compliance and work with other funders on large-scale improvements exist and should be implemented.

Journals that fail to make research discoverable breach the basic trust that researchers and their funders have in the current publishing system. Most funded researchers publish their work under the basic assumption that their journal or publisher is following best practices to ensure future use ^7^. Bodies funding health research have a responsibility to protect their investments and even more importantly, to ensure that funded research is not wasted by being published in non-credible and non-discoverable sources.

## Data availability

### Underlying data

Open Science Framework: Audit of health research funder policies and recommendations on journal publication of research: Extracted Data, https://doi.org/10.17605/OSF.IO/YUDP4 ^63^.

This project contains the following underlying data:

- Funders Data analysis data - clean 2020Apr21.dta

### Extended data

Open Science Framework: Audit of health research funder policies and recommendations on journal publication of research: Study Forms, https://doi.org/10.17605/OSF.IO/FSUQ2 ^64^

This project contains the following extended data:

- Level 1 - Searching funder websites form 2017Nov23.pdf
- Level 2 - Data extraction form 2017Nov23.pdf

Open Science Framework: Audit of health research funder policies and recommendations on journal publication of research: Protocol, https://doi.org/10.17605/OSF.IO/J6CSK ^65^

Registration of overarching OSF project: https://doi.org/10.17605/OSF.IO/Z59U6 ^66^.

Data are available under the terms of the Creative Commons Attribution 4.0 International license (CC-BY 4.0). 

## Figures and Tables

**Table:** 

#	Role	Author(s)	Definition
1	Conceptualization	LS, DM, JG	Ideas; formulation or evolution of overarching research goals and aims.
2	Data curation	LS	Management activities to annotate (produce metadata), scrub data and maintain research data (including software code, where it is necessary for interpreting the data itself) for initial use and later re-use.
3	Formal analysis	LS	Application of statistical, mathematical, computational, or other formal techniques to analyse or synthesize study data.
4	Funding acquisition	n/a – not funded	Acquisition of the financial support for the project leading to this publication.
5	Investigation	LS, KDC, MJP	Conducting a research and investigation process, specifically performing the experiments, or data/evidence collection.
6	Methodology	LS	Development or design of methodology; creation of models.
7	Project administration	LS	Management and coordination responsibility for the research activity planning and execution.
8	Resources	DM	Provision of study materials, reagents, materials, patients, laboratory samples, animals, instrumentation, computing resources, or other analysis tools.
9	Software	n/a	Programming, software development; designing computer programs; implementation of the computer code and supporting algorithms; testing of existing code components.
10	Supervision	LS, DM, JG	Oversight and leadership responsibility for the research activity planning and execution, including mentorship external to the core team.
11	Validation	LS, KDC, MJP	Verification, whether as a part of the activity or separate, of the overall replication/reproducibility of results/experiments and other research outputs.
12	Visualization	LS	Preparation, creation and/or presentation of the published work, specifically visualization/data presentation.
13	Writing – original draft	LS	Preparation, creation and/or presentation of the published work, specifically writing the initial draft (including substantive translation).
14	Writing – review & editing	LS, KDC, MJP, JCB, JG, DM, LAS, SES	Preparation, creation and/or presentation of the published work by those from the original research group, specifically critical review, commentary or revision – including pre- or post-publication stages.

## Footnotes

^1^ODA is a term coined by the Development Assistance Committee of the Organisation for Economic Co-operation and Development ( https://data.oecd.org/oda/net-oda.htm)

^2^Any European Commission funding program that explicitly stated using the European Horizon 2020 guidelines for grantees were jointly represented as “European Commission”. ( Figure 1)

^3^Beall’s List is an archived listing of potential predatory journals and publishers, as determined by librarian Jeffrey Beall between 2011 and 2017, https://web.archive.org/web/20170103170903/https://scholarlyoa.com/
