# Supplementary material for: Top health research funders’ guidance on selecting journals for funded research
Source: F1000Res. 2021 Apr 16;10:100. Originally published 2021 Feb 11. [Version 2] doi: 10.12688/f1000research.27745.2 (PMC8063518; doi:10.12688/f1000research.27745.2)
Supplement: Peer review history

# Peer review: Reviewer response for version 2

**DOI:** 10.5256/f1000research.55848.r83430

**Published:** 2021-04-19

**Authors:** Nicholas Devito

**Affiliations:** 1 The DataLab, Nuffield Department of Primary Care Health Sciences, University of Oxford, Oxford, UK

Many thanks to the authors for their consideration of my review and their revisions. While many of my comments were addressed, I do have some additional feedback based on the responses from the authors that I believe should be further considered. That said, these are all relatively minor enough that I recommend the manuscript move forward for indexing as the analysis and related discussion is overall fundamentally sound.

**-As indicated, Dr. Holmes is the CEO of a health research funding organization (not included in our sample). Her expertise of the funding landscape and whether a listing of health research funders existed or whether the chosen list was the best source of this information, was an invaluable methodological contribution to our research. We prefer not to remove this acknowledgment.**

 I still believe this reads awkwardly in the text and is not how personal communication citations are typically used. If it is just an acknowledgement it is ultimately redundant to your thanks of Dr. Holmes in the “Acknowledgements” section at the end of the piece. However this is a minor point.

**-Extraction criteria were devised de novo based on expertise within the author team. There is no reference standard for funder policies about publishing and as such we did not use any framework for extraction. We have added a clarification of this point in the methods section rather than in the discussion section.**

 The addition to the methods is appropriate although I do believe there is an argument to be made that using criteria developed in-house based on expertise, while an acceptable method, could be seen as a limitation as it is lacking further validation and consideration beyond the study team.

**-We did not apply an analytical framework to the extracted text and doing so would be a post-hoc analysis. The table/text is provided for reference/transparency of extracted data summarized in Tables 2-4. We do not feel that a summary is necessary.**

 I do not agree that a full analytic framework is required to condense multiple tables of raw verbatim text within the body of a manuscript into something more easily digestible to readers. I still strongly recommend that the authors reconsider the utility that 7 pages of Tables in the middle of the manuscript (including 4 pages just for Table 5) has for readers when Tables 2-4 summarize the extractions from this raw data as necessary for the endpoints considered and interested parties could examine the very well-curated, transparent, and clearly sign-posted raw data for themselves as necessary.

**-To be clear, we did not make a post-hoc justification regarding the lack of outreach to funders. We specifically did not have the a priori intent of verifying our data with funders. We anticipated that the results of our previous work, https://www.nature.com/news/stop-this-waste-of-people-animals-and-money-1.22554, which identified a large number of funders supporting research in predatory journals, may impact/prompt changes in funder policies once published (Sept 2017). Due to the timing of the current study, (Aug – Sept 2017), we decided not to engage funders since we did not want to influence any potential changes to their policies as a result of the study process. We did not find this to be a limitation. As expected, and as noted in our discussion, we are aware of at least one funder (NIH) who, in Nov 2017, clarified their policies about publication as a result of the previous study. The specific impacts (i.e., number and type of clarifications) of engaging funders to clarify extracted data in Goldacre et al’s previous work are unclear, however, we have added text to the limitations section outlining the additional potential benefit of engaging funders in the study process.**

 While it is true that outreach to sponsors was not specified in the protocol, it is also true that the justification for not doing so was also not provided in the protocol despite the fact that it was used in the study cited as informing your data collection/extraction methods. Therefore, while the decision not to conduct outreach was not post hoc there is no way to know if the provided justification was or not. In my original reading the provided information was not well-supported by even your own findings. Expanding on the context provided above in your revision is helpful however there is another aspect at play here. You relied on a single author for searches and only involved a second party for extractions. There is no acknowledgement of the potential that documents could have been missed by a single searcher. Outreach to the parties under investigation would allow them to confirm whether or not you have missed potentially relevant documentation on the topics of interest. It is a validation step for your methods rather than simply a way to potentially increase “uptake of our findings/ recommendations into practice.”

Is the work clearly and accurately presented and does it cite the current literature?

Partly

If applicable, is the statistical analysis and its interpretation appropriate?

Yes

Are all the source data underlying the results available to ensure full reproducibility?

Yes

Is the study design appropriate and is the work technically sound?

Yes

Are the conclusions drawn adequately supported by the results?

Yes

Are sufficient details of methods and analysis provided to allow replication by others?

Yes

Reviewer Expertise:

Metascience, health policy, publication ethics.

I confirm that I have read this submission and believe that I have an appropriate level of expertise to confirm that it is of an acceptable scientific standard.

# Peer review: Reviewer response for version 1

**DOI:** 10.5256/f1000research.30678.r79363

**Published:** 2021-03-08

**Authors:** Nicholas Devito

**Affiliations:** 1 The DataLab, Nuffield Department of Primary Care Health Sciences, University of Oxford, Oxford, UK

Many thanks for the opportunity to review this piece on health research funders’ policies on journal selection for research outputs.

 Overall, this piece investigates an interesting topic, has appropriate methods, appears well-executed, and follows open science best principles of pre-registration and open sharing of data for which it should be commended. I ultimately believe it should be recommended for indexing but a minor revision is in order. Specific points are provided below.

 Can the authors please revisit the piece for a bit of editing? The informational content all appears very relevant and well-researched but the prose can carry on at times and structurally it jumps back and forth between topics and it is difficult to connect the threads. This is most applicable to the introduction (and perhaps the Discussion as well) but I think taking a critical eye towards developing a more direct, succinct, and straight-forward writing style throughout would enhance readability. A sharper consolidation and organisation of your ideas and arguments, while trying to be more concise overall, will aid readers considerably. A brief example: “Funders ought to be concerned that funded research may be published in journals that do not ensure discoverability of content ensuring it is available to contribute to future science” could surely be consolidated to something like “Funders should ensure published research is discoverable to the scientific community.” 

 One area that doesn’t really come through clearly in the Intro is what funders get out of publication of their funded work and how this influences this dynamic. Why are they moving towards Open Access? What about other dissemination routes?

 The personal communication citation of Dr. Beverly Holmes feels a bit out of place. Is she merely stating an opinion? If so, why can the authors not just state this opinion directly themselves since I assume they share it? I’m sure Dr. Holmes is perfectly knowledgeable and her opinion is valuable, but is citing her necessary here? I’m most familiar with citations of personal communication to convey official information not published elsewhere, from a direct source, rather than simply used to state an opinion the authors agree with. The average reader won’t have any insight as to why they should trust the word of Dr. Holmes on this issue.

 The link to the protocol is upfront and clear and points readers to exactly where they can easily access more detailed information about the search/data extraction methods. Very well done by the authors.

 How were the extraction criteria derived? Did the authors base them on anything or did they come up with them de novo? This should be stated and potentially explored in the Discussion.

 One major area I was surprised that the authors did not include in their assessments is whether the funder offers to pay the open access/APCs of work they fund. This would be an important part of the dynamic of how, where, and why certain journals may be chosen and why funders should care even more about where the research they fund ends up.

 In Figure 1, it is unclear when INSERM is mentioned as to whether that is the funder being removed or the reason another funder is being removed (later context shows it’s the latter, but this is unclear). Also there is a type (“allocated”) in the “Excluded Funders n=6” box. I think ideally you would list which exact funders are entering or exiting the sample in this figure for clarity as you don’t get a good sense of what funders are actually being assessed until the results. It’s also not necessary to restate in prose everything Figure 1 already conveys (something to be cognisant of throughout).

 Table 5 is a bit overwhelming. Perhaps consider summarising each policy in Table 5 and make the full text extractions available as an appendix for interested readers.

 I don’t find the reason given for a lack of outreach to sponsors particularly compelling. The author’s state they did not verify with sponsors (despite this being a feature of the work from Goldacre *et al*. they cite as a template for this research) because “data collection took place at a time when the publishing activities...was rapidly changing.” That seems like a post hoc justification rather than an actual reason why it didn’t actually occur. It also isn’t supported by their own data as the median date of effect of policies reported was September 2014, 3 years prior to data collection and the higher IQR range is April 2016, over a year before the searches. Similarly, this article is cross-sectional, so I don’t see that reason as relevant anyway. You could simply have discarded policies from before a cutoff date, etc. Can the authors please revisit their discussion of this limitation?

 Table 8 may fit better in an appendix.

Is the work clearly and accurately presented and does it cite the current literature?

Partly

If applicable, is the statistical analysis and its interpretation appropriate?

Yes

Are all the source data underlying the results available to ensure full reproducibility?

Yes

Is the study design appropriate and is the work technically sound?

Yes

Are the conclusions drawn adequately supported by the results?

Yes

Are sufficient details of methods and analysis provided to allow replication by others?

Yes

Reviewer Expertise:

Metascience, health policy, publication ethics.

I confirm that I have read this submission and believe that I have an appropriate level of expertise to confirm that it is of an acceptable scientific standard, however I have significant reservations, as outlined above.

# Response

**Published:** 2021-04-01

**Authors:** Larissa Shamseer

**Affiliations:** Unity Health Toronto, Canada

Thank you for reviewing this work. Please see responses to your comments below.

- We have made some changes in the introduction and discussion sections to make the wording more concise. 
- We have clarified that scholarly publishing is intended to facilitate the discoverability and uptake of funded research (into practice) and that predatory journals limit this by having unreliable discovery and questionable trustworthiness. We did not set out to investigate funders’ open access (OA) policies or shifts towards OA since discoverability and other journal features compromised by predatory journals can be achieved independent of openness. We note that several sources (ROARMAP, SHERPA JULIET)  list details of funders' open access policies. This research is instead concerned with hallmarks of journal validity which have been (mis)taken as signals of research quality.
- Regarding other dissemination routes, we have clarified that this research focuses on journal publications rather than other publications routes (end of introduction section).
- As indicated, Dr. Holmes is the CEO of a health research funding organization (not included in our sample). Her expertise of the funding landscape and whether a listing of health research funders existed or whether the chosen list was the best source of this information, was an invaluable methodological contribution to our research. We prefer not to remove this acknowledgment.
- Extraction criteria were devised de novo based on expertise within the author team. There is no reference standard for funder policies about publishing and as such we did not use any framework for extraction. We have added a clarification of this point in the methods section rather than in the discussion section.
- We have added text to the introduction (2nd para) outlining the waste of research funds & APCs due to predatory journals. While we did not extract specific data on funders' APC provisions, where funders provided information about journal APC support, this can be found in Table 5.
- We have updated the suggested information in the flow diagram and left the related descriptive text in results for additional reference.
- We did not apply an analytical framework to the extracted text and doing so would be a post-hoc analysis. The table/text is provided for reference/transparency of extracted data summarized in Tables 2-4. We do not feel that a summary is necessary.
- To be clear, we did not make a post-hoc justification regarding the lack of outreach to funders. We specifically did not have the *a priori* intent of verifying our data with funders. We anticipated that the results of our previous work, https://www.nature.com/news/stop-this-waste-of-people-animals-and-money-1.22554, which identified a large number of funders supporting research in predatory journals, may impact/prompt changes in funder policies once published (Sept 2017). Due to the timing of the current study, (Aug – Sept 2017), we decided not to engage funders since we did not want to influence any potential changes to their policies as a result of the study process. We did not find this to be a limitation. As expected, and as noted in our discussion, we are aware of at least one funder (NIH) who, in Nov 2017, clarified their policies about publication as a result of the previous study. The specific impacts (i.e., number and type of clarifications) of engaging funders to clarify extracted data in *Goldacre et al’s* previous work are unclear, however, we have added text to the limitations section outlining the additional potential benefit of engaging funders in the study process.
- F1000 does not allow appendices and we have opted not to move this table to the Open Science Framework repository for this project as supplementary materials. We feel that the recommendations in this table are part of our discussion and are likely to receive little uptake if readers have to navigate away from the full text.

# Peer review: Reviewer response for version 1

**DOI:** 10.5256/f1000research.30678.r79367

**Published:** 2021-03-01

**Authors:** Shubhada Nagarkar

**Affiliations:** 1 Department of Library and Information Science, Savitribai Phule Pune University, Pune, India; 2 Centre for Publication Ethics, Savitribai Phule Pune University, Pune, Maharashtra, India

The purpose of this work is clear and the work is explicitly and precisely presented. Researchers would like to describe the policies of major health research funding agencies. The job is sound technically.

 Authors selected policies from 44 funding agencies and assessed systematically to find information and recommendations on the dissemination of research output, recommendations on criteria for the selection of journals (quality, impact factor and other metrics).

 Methodology for data collection and analysis is discussed very well and the protocol followed is described in detail.

 Statistical data can be interpreted easily. Source data can be reproducible.

 The results obtained through this exercise are well discussed and will be of interest to all funding agencies, especially those from developing countries. The findings of the study show that there is no specific guidance about how to select a journal for publication. The mention and cautionary notes on predatory publishers and their open-access nature should be made clear by these funding agencies. Authors warned policymakers about this. If the policy of these funding agencies advises researchers to current trends in publishing, researchers may not fall into pray with predatory publishers.

 Overall, this work is recommended for indexing. Similar studies can be considered for the assessment of policies of funding agencies other than health research funders.

Is the work clearly and accurately presented and does it cite the current literature?

Yes

If applicable, is the statistical analysis and its interpretation appropriate?

Yes

Are all the source data underlying the results available to ensure full reproducibility?

Yes

Is the study design appropriate and is the work technically sound?

Yes

Are the conclusions drawn adequately supported by the results?

Yes

Are sufficient details of methods and analysis provided to allow replication by others?

Yes

Reviewer Expertise:

Bibliometrics, publication ethics, institutional repositories.

I confirm that I have read this submission and believe that I have an appropriate level of expertise to confirm that it is of an acceptable scientific standard.

# Response

**Published:** 2021-04-01

**Authors:** Larissa Shamseer

**Affiliations:** Unity Health Toronto, Canada

Thank you for your thoughtful comments.
